# Toll-like 4 receptor inhibitor TAK-242 decreases neuroinflammation in rat brain frontal cortex after stress

**DOI:** 10.1186/1742-2094-11-8

**Published:** 2014-01-11

**Authors:** Iciar Gárate, Borja García-Bueno, José Luis Muñoz Madrigal, Javier R Caso, Luis Alou, María Luisa Gómez-Lus, Juan Carlos Leza

**Affiliations:** 1 Department of Pharmacology, Faculty of Medicine, University Complutense, 28040 Madrid, Spain; 2 Centro de Investigación Biomédica en Salud Mental (CIBERSAM), 28040 Madrid, Spain; 3 Instituto de Investigación Sanitaria Hospital 12 de Octubre and Instituto Universitario de Investigación en Neuroquímica UCM, 28040 Madrid, Spain; 4 Department of Psychiatry, Faculty of Medicine, University Complutense, 28040 Madrid, Spain; 5 Department of Medicine – Microbiology Area, Faculty of Medicine, University Complutense, 28040 Madrid, Spain

**Keywords:** Restraint stress, TLR-4 signalling, Bacterial translocation, Neuroinflammation, Frontal cortex

## Abstract

**Background:**

The innate immune response is the first line of defence against invading microorganisms and it is also activated in different neurologic/neurodegenerative pathological scenarios. As a result, the family of the innate immune toll-like receptors (TLRs) and, in particular, the genetic/pharmacological manipulation of the TLR-4 signalling pathway emerges as a potential therapeutic strategy. Growing evidence relates stress exposure with altered immune responses, but the precise role of TLR-4 remains partly unknown.

**Methods:**

The present study aimed to elucidate whether the elements of the TLR-4 signalling pathway are activated after acute stress exposure in rat brain frontal cortex and its role in the regulation of the stress-induced neuroinflammatory response, by means of its pharmacological modulation with the intravenous administration of the TLR-4 specific inhibitor TAK-242. Considering that TLR-4 responds predominantly to lipopolysaccharide from gram-negative bacteria, we checked whether increased intestinal permeability and a resultant bacterial translocation is a potential regulatory mechanism of stress-induced TLR-4 activation.

**Results:**

Acute restraint stress exposure upregulates TLR-4 expression both at the mRNA and protein level. Stress-induced TLR-4 upregulation is prevented by the protocol of antibiotic intestinal decontamination made to reduce indigenous gastrointestinal microflora, suggesting a role for bacterial translocation on TLR-4 signalling pathway activation. TAK-242 pre-stress administration prevents the accumulation of potentially deleterious inflammatory and oxidative/nitrosative mediators in the brain frontal cortex of rats.

**Conclusions:**

The use of TAK-242 or other TLR-4 signalling pathway inhibitory compounds could be considered as a potential therapeutic adjuvant strategy to constrain the inflammatory process taking place after stress exposure and in stress-related neuropsychiatric diseases.

## Background

The innate immune response is the first line of host defence against invading microorganisms [1]. It is also specifically activated both at the peripheral and central nervous system (CNS) level in different neurologic/neurodegenerative pathological scenarios (that is, stroke, multiple sclerosis and Alzheimer’s disease) [2]. The activation of the innate immune system in these CNS disorders presents an apparent “doubled edged sword” potential, exerting either beneficial or detrimental effects that clearly warranted further investigation to manage its proper pharmacological modulation for therapeutic purposes [3].

The main agents of the innate immune response are the family of toll-like receptors (TLRs). TLRs are pattern recognition receptors that detect circulating pathogen-associated molecular patterns (PAMPs), which are found in pathogens but not in mammalian cells. These patterns, such as the bacterial lipopolysaccharide (LPS), trigger a complex inflammatory cascade, characterized by the production of cytokines, enzymes and other inflammatory mediators, and the activation of oxidative and nitrosative stress pathways that can have an impact on several aspects of the CNS homeostasis and pathology [4,5].

TLRs are highly expressed in immune cells in response to pathogens, a variety of cytokines and environmental stressors [6]. In the last years, TLR expression has been found in different CNS intrinsic cell types, such as neurons, astrocytes, resident microglia, or cells in cerebral microvasculature, plexus choroideus and leptomeninges [7]. This ubiquitous expression of the TLR family has challenged the role of the immune innate system in the brain and, as a result, the view of the brain as an immune privileged organ has been re-evaluated. In addition, this extended distribution could suggest other roles for TLRs in non-infectious CNS diseases/injury, recognizing a number of endogenous molecules released from damaged tissues (damage-associated molecular patterns, or DAMPs) [8] in a great diversity of processes, such as autoimmunity, neurogenesis, brain plasticity and behaviour [7,9,10].

The most studied member of the TLR family is TLR-4, which predominantly responds to LPS from Gram-negative bacteria [11] through its co-receptor, myeloid differentiation protein-2 (MD-2), a requisite for LPS signalling of TLR-4 [12]. To achieve specificity of signalling, TLR-4 recruits some other adapter proteins during intracellular signal transduction, such as the myeloid differentiation factor 88 (MyD88). After various consecutive steps in the transduction pathway (that is, specific kinases), the activation of the prototypic inflammatory nuclear transcription factor NF-κB [13] culminates in the production of NF-κB-dependent pro-inflammatory mediators such as, for example, the enzymes inducible nitric oxide synthase (iNOS) and inducible cyclooxygenase (COX-2) [14].

In the last years, increasing attention has been paid to the potential role of the immune system in the pathophysiology of stress-related neuropsychiatric diseases such as major depression or chronic fatigue syndrome [15,16]. In many cases, both depression and chronic stressors have been associated with decreased acquired immunity and an increased susceptibility to infections and inflammation, with important general consequences for health [17]. However, in the last decade it has been demonstrated that after stress exposure or during certain episodes of depression an inflammatory/immune innate response is strongly activated [15]. Thus, inflammatory cellular pathways are activated in particular brain areas (that is, the frontal cortex (FC)) after stress exposure, with a key role for NF-κB as a crucial factor in the oxidative/nitrosative damage produced [18]. On the other hand, NF-κB activation has also been related to neuronal plasticity and survival, depending on the activated cell type [19].

The precise role of TLR-4 in the inflammatory responses activated after stress exposure in the brain and periphery are still a matter of debate, as well as the regulatory mechanism(s) implicated. Remarkably, some models of stress show increased intestinal permeability and a resultant bacterial translocation to the systemic circulation and/or other organs such as the liver, spleen and mesenteric lymph nodes [20]. These circulating Gram-negative bacteria are a major source of LPS, which can activate brain TLR-4 through multiple pathways, inducing a neuroinflammatory response. This proposed mechanism known as “leaky gut” also takes place in patients with depression or chronic fatigue syndrome and it has been related to the inflammatory pathophysiology of the diseases [16,21]. However, the molecular pathways affected may vary between pathologies: in depression, there are significant associations between bacterial translocation and increased oxidative/nitrosative stress pathways [16], while in chronic fatigue syndrome bacterial translocation is accompanied by increased levels of cytokines [21].

In a stress-related scenario, TLR-4 has been presented as a specific regulator of the adrenal response to inflammatory stimuli [22], acute and chronic stress exposure included [23,24].

Taking into account all this background, the present study aimed to evaluate whether the TLR-4 signalling pathway is activated after acute stress exposure in rat FC and the potential regulatory mechanisms implicated (that is, bacterial translocation).

Moreover, the possible role of TLR-4 in the regulation of stress-induced neuroinflammation was also analyzed by means of TLR-4 pharmacological modulation with its specific inhibitor TAK-242.

## Methods

### Animals

Male outbred Wistar Hannover rats (HsdRccHan:Wist, from Harlan, Spain), initially weighing 200 to 225 g, were housed five per cage and maintained in an animal holding room controlled at a constant temperature of 24 ± 2°C with a relative humidity of 70 ± 5% and a 12 hour light–dark cycle (lights on at 08:00). Animals were fed a standard pellet chow (standard rat chow A04 SAFE, Scientific Animal Food and Engineering, Augy, France) with free access to fresh tap water and were maintained under constant conditions for 7 days prior to stress. All experimental protocols followed the guidelines of the Animal Welfare Committee of the Universidad Complutense according to European legislation (2003//65/ EC).

### Experimental design

First, in order to include a positive control to directly test the connection between infection and upregulation of TLR-4 in rat brain FC, a group of rats was injected intraperitoneally (i.p.) with 500 μg/kg LPS and sacrificed 2 hours later. A correspondent control group was included (Control).

Second, in order to verify the time course of TLR-4 activation after stress exposure, three different groups of animals (n = 6 per group) were respectively exposed to 2 hours (S2h), 6 hours (S6h) and 4 days (6 hours/day for 4 days) (S4d) of restraint stress. A correspondent control group was included (Control).

Next, in order to explore the possibility of Gram-negative LPS induction of TLR-4 caused by intestinal bacterial translocation after stress, stressed animals were treated with antibiotic (STRESS + ATB) or its vehicle (STRESS).

In order to clarify the direct implication of the TLR-4 pathway in the stress-induced neuroinflammation, two groups of animals were intravenously (i.v.) injected with the TLR-4 inhibitor TAK-242 (S(TAK)) or vehicle (STRESS) and then submitted to 6 hours of restraint stress.

None of the parameters studied were modified in the two different vehicle-treated groups of rats when compared with the non-injected animals. To simplify figures, both groups were unified into one (STRESS).

### Pharmacological tools

TAK-242 (ethyl (6R)-6-[N-(2-chloro-4-fluorophenyl)sulfamoyl]cyclohex-1-ene-1-carboxylate) [25] is a specific inhibitor of TLR-4 that works by blocking its intracellular domain TIR Toll/IL-1 Receptor [26] without affecting the extracellular docking with its main ligand LPS [27]. Thus, TAK-242 inhibits the intracellular signalling of TLR-4, preventing the binding to its adapter molecules [28].

TAK-242 was i.v. injected in the tail vein at a dose of 0.5 mg/kg immediately after (approximately 10 seconds) introducing the animal to the plastic restrainer. This dose was chosen on the basis of previous *in vivo* studies reporting its anti-inflammatory/antioxidant and neuroprotective profile in microglia exposed to hypoxia [29]. Dimethyl sulphoxide at a concentration of 0.9% was used as vehicle.

### Intestinal antibiotic decontamination

We followed a previously described protocol for rats [30]. Briefly, animals were given drinking water *ad libitum* containing streptomycin sulphate (2 mg/ml) and penicillin G (1,500 U/ml), 5 days before the first session of stress (at 08:00) until the moment of sacrifice, to reduce indigenous gastrointestinal microflora. The amount of antibiotic consumed was >75% of that initially administrated. To discard a possible effect of the antibiotic treatment on the immune/inflammatory response, the levels of NF-κB, iNOS and COX-2 in control and LPS (0.5 mg/kg i.p.) rats with and without antibiotic were checked and no major changes were found between the different groups (data not shown).

### Stress protocol and tissue samples

The restraint stress protocol was performed using a plastic rodent restrainer that allowed for a close fit to rats starting in all groups at 09:00 [31]. Control animals were not subjected to stress, but were handled at 09:00 for a few seconds, and food and water were removed during the period of time that the stressed rats were kept in the restrainer.

Animals were killed immediately after restraint using sodium pentobarbital (320 mg/kg, i.p.; Vetoquinol, Madrid, Spain). The lethal injection of sodium pentobarbital was performed when the animal was still in the plastic restrainer at the end of the stress period. After decapitation, the brain was removed from the skull, and after careful removal of the meninges and blood vessels, the frontal cortical areas from both brain hemispheres were excised and frozen at -80ºC until assayed. Peripheral leukocytes express TLR-4 and could be a significant source of pro-inflammatory mediators after stress exposure. To manage this possible confounding factor, a group of stressed animals was transcardially saline-perfused prior to collection of brain tissue. This group of animals did not present differences in TLR-4, iNOS and COX-2 expression in brain homogenate samples compared to the group of stressed animals without prior saline perfusion.

Rat brain FC was chosen because of its high levels of pro-inflammatory/anti-inflammatory mediators, its susceptibility to the neuroinflammatory process elicited by stress [17] and finally because this brain area is an important neural substrate for the regulation of the hypothalamo-pituitary-adrenal axis response to stress [32].

### Preparation of nuclear extracts

A modified procedure based on the method of Schreiber and colleagues [33] was used: tissues (brain FC) were homogenized in 300 μl buffer (10 mmol/l *N*-2-hydroxyethylpiperazine-N-2-ethanesulfonic acid (pH 7.9); 1 mmol/l EDTA, 1 mmol/l EGTA, 10 mmol/l KCl, 1 mmol/l dithiothreitol, 0.5 mmol/l phenylmethylsulfonyl fluoride, 0.1 mg/ml aprotinin, 1 mg/ml leupeptin, 1 mg/ml Na-*p*-tosyll-lysine-chloromethyl ketone, 5 mmol/l NaF, 1 mmol/l NaVO4, 0.5 mol/L sucrose, and 10 mmol/l Na2MoO4). After 15 minutes, Nonidet P-40 (Roche, Mannheim, Germany) was added to reach a 0.5% concentration. The tubes were gently vortexed for 15 seconds, and nuclei were collected by centrifugation at 8000 *g* for 5 minutes. Supernatants were considered as the cytosolic fraction. The pellets were resuspended in 100 μl buffer supplemented with 20% glycerol and 0.4 mol/l KCl and gently shaken for 30 minutes at 4ºC. Nuclear protein extracts were obtained by centrifugation at 13,000 *g* for 5 minutes, and aliquots of the supernatant were stored at -80ºC. All steps of the fractionation were carried out at 4ºC.

### Bacterial translocation

The abdominal skin was shaved and sterilized with an iodine solution. After blood sampling, mesenteric lymph nodes (MLNs) were removed under sterile conditions. After weighing and homogenization, aliquots (2 ml) of serial 10-fold dilutions of the suspension were plated onto 5% blood and McConkey’s agar plates for recovery of aerobic bacteria, and Brucella blood agar plates supplemented with vitamin K_1_ and hemin for anaerobic bacteria. After 24 and 48 hours incubation at 37°C for aerobic and anaerobic cultures, respectively, colonies were counted [18]. Quantitative culture results were expressed as the number of colony-forming units (CFU) per mg tissue. Any positive MLN cultures were considered indicative of bacterial translocation from the intestinal lumen. Bacterial strain was identified by Gram stain, biochemical tests and standard biochemical identification systems.

In addition, a piece of tissue from the left hepatic lobule was obtained and immediately frozen for the posterior determination of the lipopolysaccharide binding protein (LBP) mRNA levels. LBP is a soluble acute-phase protein that binds to LPS to elicit immune responses by presenting the LPS to TLR-4. The hepatic tissue was used for this determination because it is one of the main organs where LBP protein synthesis is increased in acute phase responses against LPS [34].

### Western blot analysis

Brain frontal cortices were used to determine expression levels of the oxidative/nitrosative and inflammatory enzymes iNOS and COX-2, the inflammatory transcription factor NF-κB (p65 subunit), and TLR-4 and its adapter proteins MyD88 and MD-2. In the case of the NF-κB subunit p65, the analysis was carried out in nuclear extracts from FC samples; for the inhibitory protein of NF-κB, IκBα, cytosolic extracts were used (see previous point).

After adjusting protein levels in the resultant supernatants, homogenates were mixed with Laemmli sample buffer (BioRad, Hercules, CA, USA) and 10 μl (1 mg/ml) were loaded into an electrophoresis gel. Next, the membranes were blocked in 10 mM Tris-buffered saline containing 0.1% Tween-20 and 5% skim milk/BSA, then the membranes were incubated with specific primary antibodies: from Santa Cruz Biotechnology (CA, USA) against iNOS (rabbit polyclonal antibody raised against a peptide mapping at the amino terminus of iNOS of human origin in a dilution of 1:1000 in TBS-Tween) (sc-651); COX-2 (goat polyclonal antibody raised against a peptide mapping at the C-terminus of COX-2 of mouse origin in a dilution of 1:750 in 5% BSA in TBS-Tween) (sc-1747); NF-κB p65 subunit (rabbit polyclonal NF-κB p65 raised against an epitope mapping within the N-terminus of NF-κB p65 of human origin in a dilution of 1:500 in BSA 2%) (sc-109); TLR-4 (goat polyclonal antibody raised against an epitope mapping within an extracellular domain of TLR4 of mouse origin in a dilution of 1:1000 in BSA 2%) (sc-16240); MD-2 (rabbit polyclonal antibody raised against an epitope corresponding to amino acids 1–160 representing full length MD-2 of human origin in a dilution of 1:1000 in BSA 2%) (sc-20668); from Abcam (Cambridge, UK) against MyD88 (rabbit polyclonal antibody raised against amino acids 279–296 of MyD88 of human origin in a dilution of 1:1000 in BSA 2%) (ab-2064); and from R&D systems (Abingdon, UK) against 4-hydroxynonenal (4-HNE) adducts of histidine residues (monoclonal raised against HLH-coupled 4-HNE in a dilution of 1:1000 in BSA 5%) (MAB3249). After washing with 10 mM Tris-buffered saline containing 0.1% Tween-20, the membranes were incubated with the respective horseradish peroxidase-conjugated secondary antibodies for 90 minutes at room temperature. Blots were imaged using an Odyssey® Fc System (Li-COR Biosciences, Lincoln, Nebraska USA) and were quantified by densitometry (NIH ImageJ® software, National Institutes of Health, Bethesda, Maryland, USA). Densitometric data are expressed in arbitrary units of optical density. In all Western blot analyses, the housekeeping gene β-actin was used as loading control except for the case of the NF-κB p65 subunit in which the loading control was the nuclear factor SP1 (blots shown in the respective figures).

### Real time-PCR analysis

Total cytoplasmic RNA was prepared from samples of FC using TRIZOL® reagent (Invitrogen, Life Technologies, Carlsbag, CA, USA) (following the TRIZOL® datasheet); aliquots were converted to cDNA using random hexamer primers. Semi-quantitative changes in mRNA levels were estimated by real time-PCR (RT-PCR).

Semi-quantitative changes in mRNA levels were estimated by using the following cycling conditions: 35 cycles of denaturation at 95ºC for 10 seconds, annealing at 58 to 61ºC for 15 seconds depending on the specific set of primers, and extension at 72ºC for 20 seconds. Reactions were carried out in the presence of SYBR green (1:10000 dilution of stock solution from Molecular Probes, Eugene, OR, USA), carried out in a 20-l reaction in a Rotor-Gene (Corbett Research, Mortlake, NSW, Australia). The primers used were for TLR-4: forward: 5′-AAC CAG CTG TAT TCC CTC AGC ACT-3′ and reverse: 5′-ACT GCT TCT GTT CCT TGA CCC ACT-3′; for MD-2: forward: 5′-CTC CGA TGC AAT TAT TTC CTA C -3′ and reverse: 5′-TGG CAC AGA ACT TCC TTA CG-3′; for MyD88: forward: 5′-TAA GTT GTG TGT GTC CGA CCG TGA-3′ and reverse: 5′-ATC AGT CGC TTC TGT TGG ACA CCT-3′; for iNOS: forward: 5′-CTG CTG GTG GTG ACA AGC ACA TTT-3′ and reverse: 5′-ATG TCA TGA GCA AAG GCG CAG AAC-3′; for COX-2: forward: 5′-ACT GGG CCA TGG AGT GGA CTT AAA-3′ and reverse: 5′-AAC TGC AGG TTC TCA GGG ATG TGA-3′; for IL-1β: forward: 5′- ACC TGC TAG TGT GTG ATG TTC CCA-3′ and reverse 5′- AGG TGG AGA GCT TTC AGC TCA CAT-3′; and finally for LBP: forward: 5′- TGA CAT GTT ACC GCC TGA CTC CAA -3′, reverse: 5′- AGA CCA CTG TTC CAA GAA GCT CCA -3′. Relative mRNA concentrations were calculated from the take-off point of reactions using included software, and tubulin primer levels were used to normalize data.

### Perfusion and histology

At the end of the last session of stress, rats were anaesthetized and perfused via the ascending aorta with 4% paraformaldehyde in 0.1 M PBS buffer, pH 7.4, and the brains were removed, overnight postfixed, and cryoprotected in 15% sucrose for 24 hours. Regularly spaced series of 30-μm thick coronal sections were collected in cryoprotectant solution and stored at -20°C until processing.

### Immunohistochemistry

To identify the cell type(s) displaying TLR-4-like immunoreactivity, a dual immunofluorescence protocol was used. Sections were incubated with antisera for TLR-4 (antibody already described in Western Blot analysis section (1:500) and either/or (a) a rabbit polyclonal Anti-NeuN Alexa Fluor® 488 Conjugate (Millipore Ibérica, Madrid, Spain; ABN78A4, 1:3000), used here as marker for neurons; (b) a rabbit polyclonal anti-ionized calcium binding adaptor molecule 1 (IBA1) (WAKO Pure Chemical Industries Ltd. #019-19741, 1:3000), used here as a marker for parenchymal microglia; (c) a mouse monoclonal anti-glial fibrillary acidic protein (GFAP)-like astrocyte marker (610566, BD Transduction Laboratories, San Jose, CA, USA).

The respective primary antisera were incubated for 48 hours at 4°C. Subsequently, the sections were incubated for 1 to 2 hours at room temperature with Alexa 555-conjugated donkey anti-goat IgG (1:1000; Molecular Probes/Invitrogen) to localize TLR-4 and with Alexa 488-conjugated donkey anti-rabbit IgG (1:2000; Molecular Probes/Invitrogen) for NeuN, IBA1 and GFAP, respectively. Samples were mounted using Prolong Gold antifade reagent with DAPI (Life Technologies). Control experiments included incubation of tissue sections from control and stressed animals with each antiserum singly and then with both secondary antisera to ensure that the latter did not cross-react with the inappropriate primary antiserum or with each other. Imaging was performed using a Leica SP2 TCS AOBS spectral confocal microscope (Wetzlar, Germany).

### NF-κB transcription factor assay

NF-κB transcription factor activity was determined on nuclear extracts using an ELISA-based kit, which allows detecting and quantifying the specific transcriptional activity of NF-κB (Cayman Chemicals, Tallin, Estonia).

Briefly, nuclear extracts were incubated in a multiwell plate coated with specific NF-κB p65 subunit response element probes, and p65 bounded to its response element probe was detected using a specific antibody against this subunit. Horseradish peroxidase-labelled secondary antibody was added and the binding was detected by spectrophotometry. Measurement was performed according to the manufacturer’s instructions. This assay is specific for p65 activation, and it does not cross-react with other NF-κB subunits, such as p50.

### Lipid peroxidation

Lipid peroxidation was measured by the thiobarbituric acid test [35] with some modifications. Cerebral cortex was sonicated in 10 volumes 50 mmol/l phosphate buffer and deproteinized with 40% trichloroacetic acid and 5 mol/l HCl, followed by the addition of 2% (wt/vol) thiobarbituric acid in 0.5 mol/l NaOH. The reaction mixture was heated in a water bath at 90°C for 15 minutes and centrifuged at 12000 *g* for 10 minutes. The pink chromogen was measured at 532 nm in a Beckman DU-7500 spectrophotometer (Beckman Coulter, Brea, CA, USA). The results were expressed as nmol/mg of protein.

### Protein assay

Protein levels were measured using the Bradford method based on the principle of protein-dye binding [36].

### Chemicals

Unless otherwise stated, the chemicals were from Sigma Spain, Madrid.

### Statistical analyses

Data in text and figures are expressed as mean ± SEM. For multiple comparisons a one-way analysis of variance followed by the Newman–Keuls *post hoc* test to compare all pairs of means between groups was made. When comparing only two experimental groups a two-tailed t-test was employed. A *P* value <0.05 was considered statistically significant.

## Results

### Expression of toll-like receptor-4 signalling pathway elements in the brain frontal cortex of rats submitted to stress

**Figure 1:**
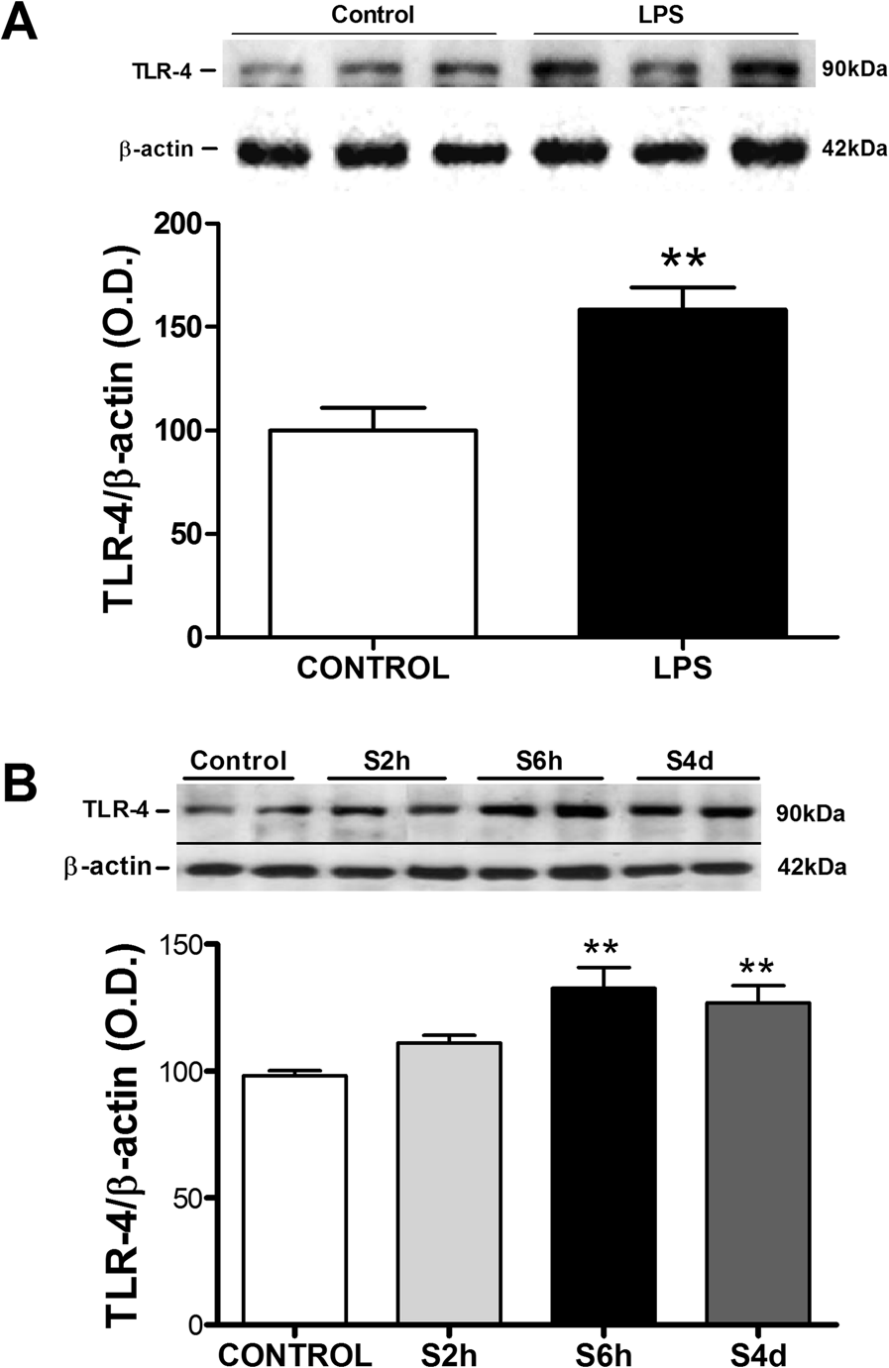
**Time course of toll-like receptor-4 expression after lipopolysaccharide and restraint stress exposure.** Protein levels of toll-like receptor (TLR)-4 in brain frontal cortex samples of **(A)** control and lipopolysaccharide-treated rats (LPS; 500 μg/kg), and **(B)** stressed rats during 2 hours (S2h), 6 hours (S6h) and 4 days (2 hours per day 4 days (6 hours per day)) (S4d). Data are representative of two experiments (n = 3 to 4 per group in each experiment). Two-tailed t-test **(A)** and one-way analysis of variance followed by Newman–Keuls *post hoc* test **(B)**. **P < 0.01 versus control. O.D., optical density.

The i.p. administration of LPS produced a significant increase in TLR-4 levels in the FC (Figure 1A). This result suggests that an increase in LPS systemic levels, mimicking an infection, was capable of producing alterations in TLR-4 expression in this specific brain area.

In order to elucidate whether some elements of the TLR-4 signalling cascade that activates NF-κB are also upregulated in rat FC after stress exposure, we then studied the expression at the mRNA and protein level of TLR-4, MD-2 and MyD88. In this way, a time course of TLR-4 expression after 2 hours, 6 hours and 4 days (6 hours/day) of restraint stress was made. After 2 hours of stress exposure, TLR-4 protein expression was not increased but peaked at 6 hours and remained elevated after 4 days (6 hours/day) of stress (Figure 1B). Complementary studies using quantitative PCR also showed increased levels of TLR-4 mRNA after 6 hours of stress exposure (Figure 1B).

Based on these temporal results, we decided to study the rest of the parameters after 6 hours of restraint stress exposure.

### Cellular types displaying toll-like receptor-4 immunoreactivity in brain frontal cortex

**Figure 2:**
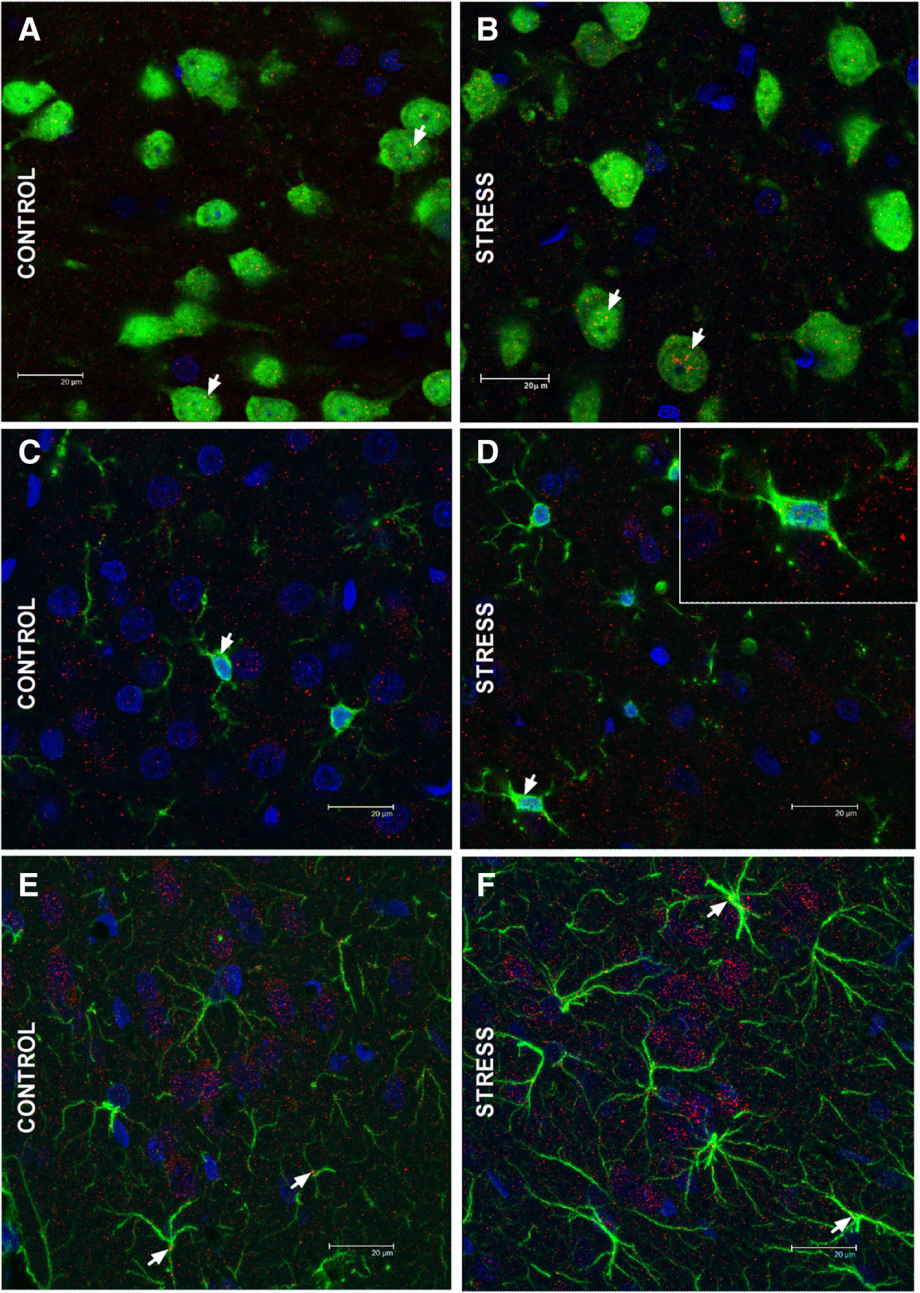
**Toll-like receptor-4 immunoreactivity in the brain frontal cortex of rats in control and stress conditions.** Sections (30 μm) through the frontal cortext of rats in **(A, C, E)** control and **(B, D, F)** stress conditions. The respective cellular markers appear in green: neurons are identified with NeuN **(A, B)**, microglia with IBA-1 **(C, D)** and astroglia with glial fibrillary acidic protein **(E, F)**. In all cases toll-like receptor (TLR)-4 is marked in red. TLR-4 localizes to the respective cellular marker with some areas of overlap appearing yellow/orange in the merged image (white arrows in **A-F**). Scale bars: 20 μm.

A qualitative approach trying to identify the cellular types where TLR-4 is expressed in brain FC was made. A detailed examination of the images indicates that TLR-4 is expressed predominantly in neurons (Figure 2A, B) and at very low levels in microglia (Figure 2C, D) and astroglia (Figure 2E, F) both in control and after stress exposure conditions. TLR-4 immunoreactivity in neurons is uniformly distributed in the neuronal soma. In microglia, the localization of TLR-4 is perinuclear. In the case of astroglia, TLR-4 immunostaining is slightly present in astrocyte somata. In addition, no major differences in TLR-4 expression or cellular distribution were found between control and stressed rats in the three cellular types studied.

### Possible regulatory mechanisms of toll-like receptor-4 activation in brain frontal cortex after stress

**Figure 3:**
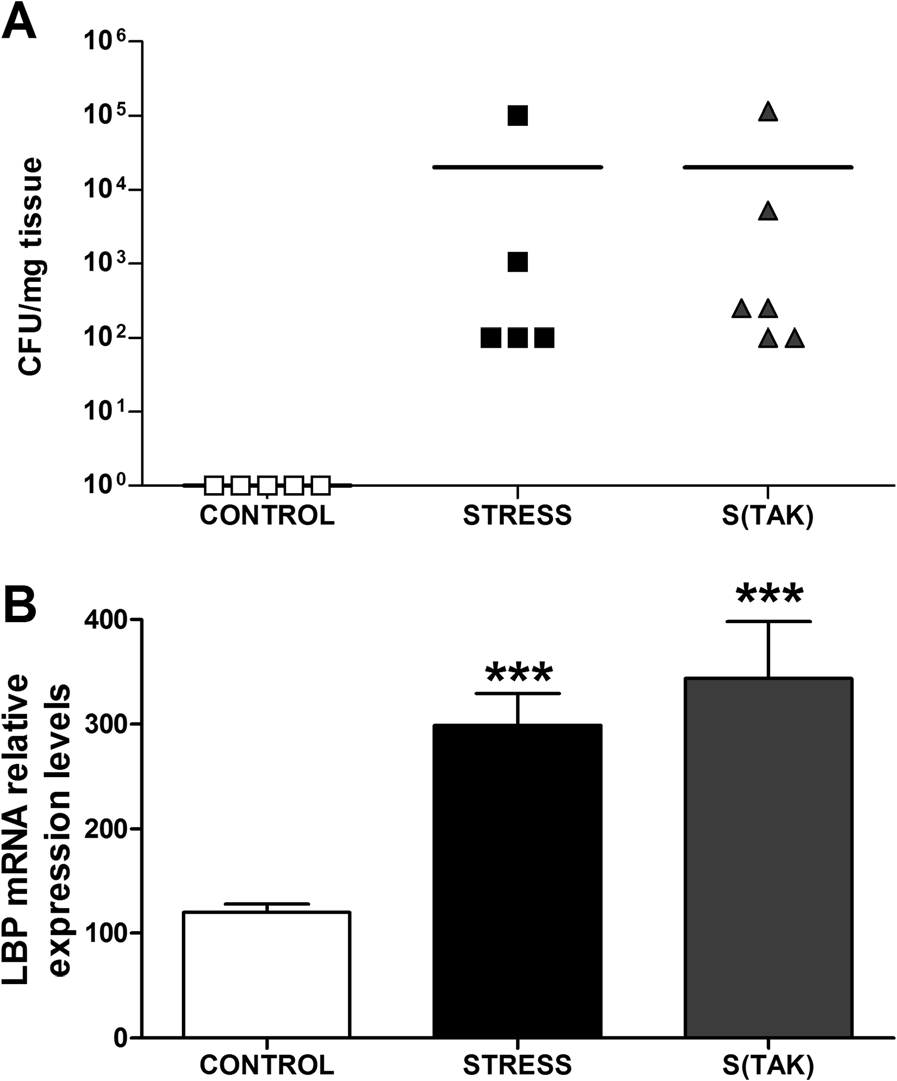
**Bacterial translocation after acute stress exposure.** Effects of TAK-242. **(A)** Bacteria colony-forming units (CFUs) per mg of tissue detected in mesenteric lymph nodes of control (CONTROL) and stressed rats with TAK-242 (S(TAK)) or without TAK-242 (STRESS). The data represent the CFU/mg found in each animal. **(B)** Lipopolysaccharide binding protein (LBP) hepatic mRNA levels of control and stressed rats with or without TAK-242 (S(TAK) and STRESS, respectively). mRNA data are normalized by tubulin. Data represent the mean ± SEM (n = 7 to 8 per group). One-way analysis of variance followed by Newman–Keuls *post hoc* test. ***P < 0.05 versus control.

TLR-4 activation by LPS switches on intracellular inflammatory pathways. In order to clarify the origin of the stress-induced activation of the TLR-4 pathway, we studied the bacterial translocation in MLNs, as well as LBP mRNA levels in the liver. The group of stressed animals showed a consistent presence of viable bacterial CFU per mg of tissue in their MLNs, and an increase in hepatic LBP mRNA levels compared to the control group (Figure 3).

The qualitative analysis identified the Gram-positive bacterial strains *Peptostreptococcus*, *Actinomyces*, *Micrococcus, Leuconostoc* and *Lactobacillus* sp*.* and the Gram-negative *Escherichia coli, Proteus mirabilis, Enterobacter agglomerans, Porphyromonas* and *Bacteroides fragilis* in the MLNs of stressed rats submitted to 6 hours of restraint. All these bacterial strains are part of the resident intestinal microbiota of Wistar rats.

### Effects of intestinal decontamination on stress-induced bacterial translocation and toll-like receptor-4 activation

**Figure 4:**
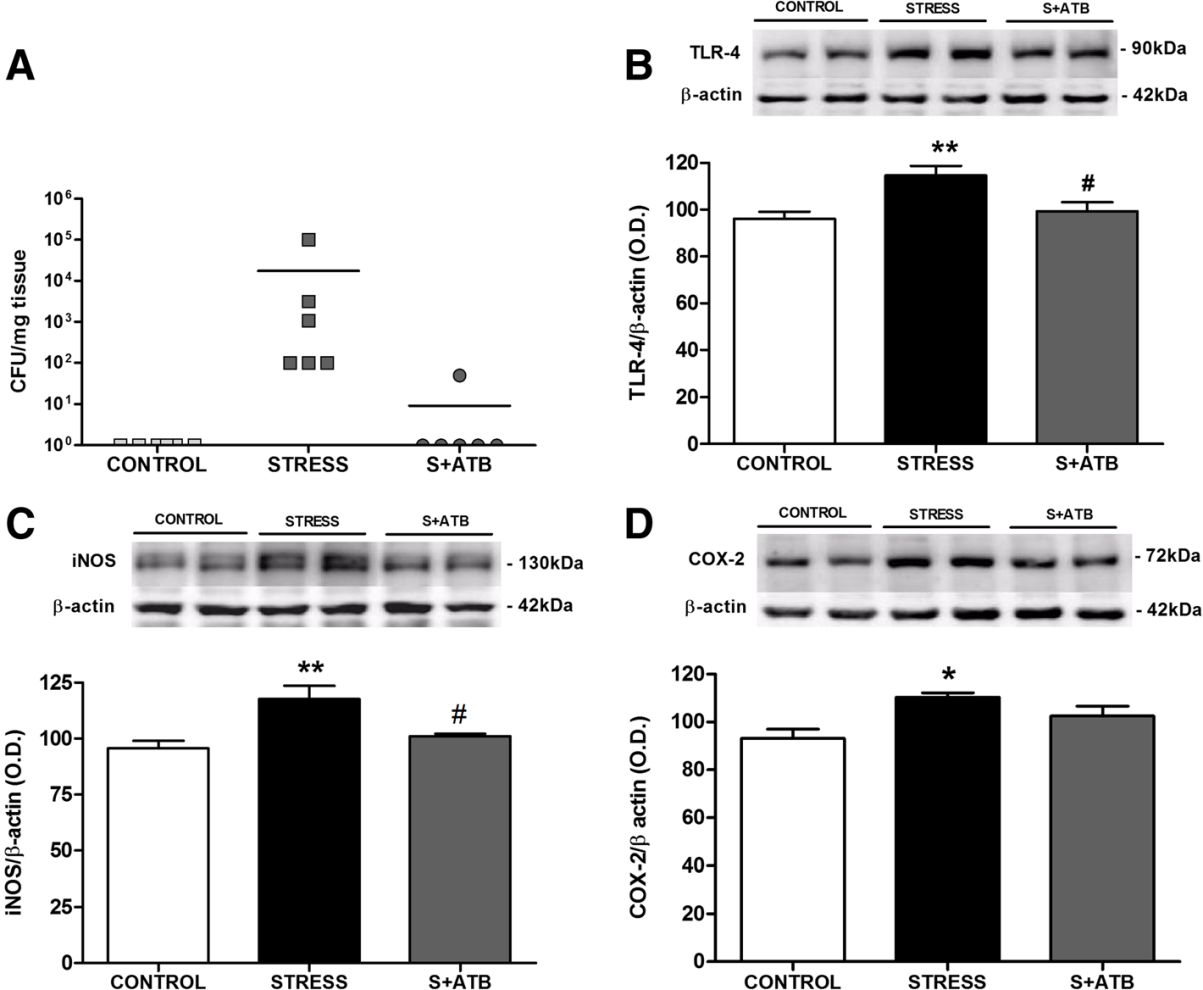
**Intestinal decontamination prevents stress-induced bacterial translocation and toll-like receptor-4 signalling pathway activation. (A)** Bacteria colony-forming units (CFUs) per mg of tissue detected in mesenteric lymph nodes in control (CONTROL) and stressed rats with (S + ATB) or without (STRESS) antibiotic treatment. The data represent the CFU found in each animal (n = 6 per group). **(B)** Toll-like receptor (TLR)-4, **(C)** inducible nitric oxide synthase (iNOS) and **(D)** inducible cyclooxygenase (COX-2) protein levels (Western blot) in brain frontal cortex samples of control and stressed rats with or without antibiotic treatment (S + ATB and STRESS, respectively). For TLR-4, the data are representative of two experiments (n = 2 to 3 per group in each experiment). One-way analysis of variance followed by Newman–Keuls *post hoc* test. *P < 0.05 versus control; **P < 0.01 versus control; ^#^*P* < 0.05 versus stress. O.D., optical density.

Based on the previous results, we carried out an experiment using antibiotic intestinal decontamination in an attempt to directly demonstrate the role of bacterial translocation on TLR-4 signalling pathway activation in the brain FC after acute restraint stress exposure. We could not detect any bacterial translocation in the MLNs of rats treated with antibiotic (Figure 4A). In addition, the blocking effect of bacterial decontamination is extended to stress-induced TLR-4, iNOS and COX-2 (Figure 4B-D) overexpression in brain FC.

### TAK-242 effects on stress-induced toll-like receptor-4 activation in brain frontal cortex

**Figure 5:**
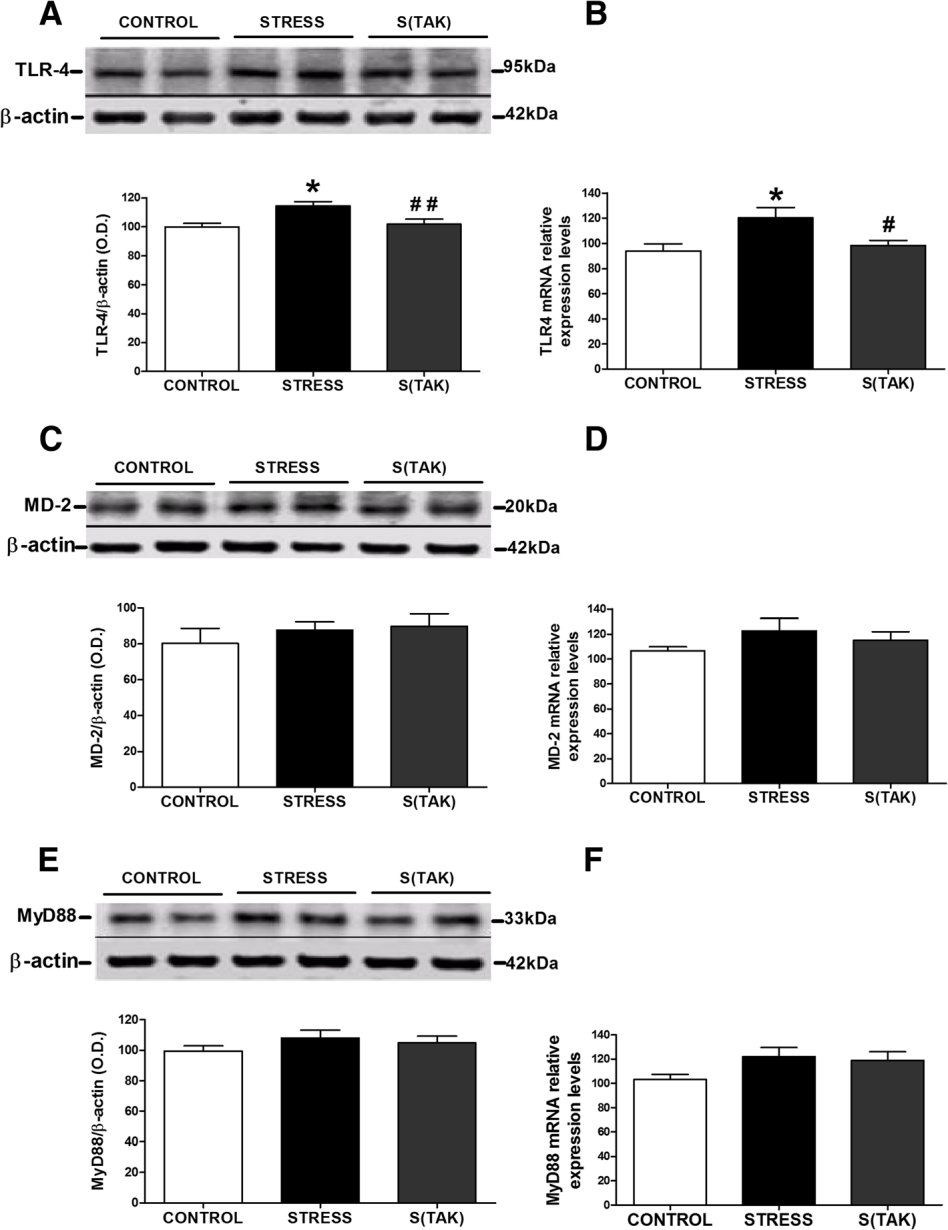
**Toll-like receptor-4 upregulation is elicited by acute restraint stress exposure in rat brain frontal cortex.** Protein levels of **(A)** toll-like receptor (TLR)-4, **(C)** myeloid differentiation protein-2 (MD2) and **(E)** myeloid differentiation factor 88 (MyD88) in brain frontal cortex samples of control (CONTROL) and stressed rats with (S(TAK)) or without (STRESS) TAK-242. The densitometric data of the respective band of interest are normalized by β-actin (lower band). Data are representative of two experiments (n = 3 per group in each experiment). O.D., optical density. mRNA relative levels of **(B)** TLR-4, **(D)** MD2 and **(F)** MyD88 in brain frontal cortex samples of control and stressed rats with or without TAK-242 (S(TAK) or STRESS, respectively). mRNA data are normalized by tubulin. Data represent the mean ± SEM (n = 4 to 5 per group). One-way analysis of variance followed by Newman–Keuls *post hoc* test. *P < 0.05 versus control; ^#^*P* < 0.05 versus stress. ^##^*P* < 0.01 versus stress. This statistical data can be used for A and B.

TAK-242 i.v. administration at the beginning of the stress session completely blocked TLR-4 mRNA and protein upregulation after stress exposure (Figure 5A, B). On the contrary, MD-2 and Myd88 expression remained unaltered after 6 hours of stress exposure (Figure 5C-F). In addtion, TAK-242 did not affect MD-2 and MyD88 expression in any conditions (Figure 5C-F).

### Effects of TAK-242 on stress-induced bacterial translocation

In order to clarify whether TAK-242 administration was able to modify the bacterial translocation elicited by stress exposure, we compared the bacterial translocation in MLNs and the LBP hepatic levels in control and stressed animals with/without i.v. pre-administration of TAK-242. Both stressed groups of animals (± TAK-242) showed a similar presence of viable bacterial colony forming units (CFU) per mg in their MLNs, and increased hepatic LBP mRNA levels compared to their respective control groups (Figure 3).

### Role of toll-like receptor-4 in stress-induced neuroinflammation and oxidative/nitrosative mediator over-accumulation in rat brain frontal cortex

**Figure 6:**
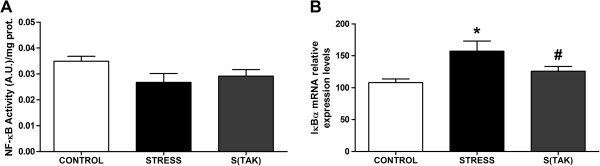
**TAK-242 effects on NF-κB signalling under control and stress conditions. (A)** Transcriptional activity of NF-κB p65 subunit in nuclear extracts of control (CONTROL) and stressed rats with TAK-242 (S(TAK)) or without TAK-242 (STRESS). The data represent the mean ± SEM (n = 5 per group). One-way analysis of variance followed by Newman–Keuls *post hoc* test. A.U., arbitrary units. **(B)** IκBα mRNA relative levels in brain frontal cortex samples from control and stressed rats with or without TAK-242 (S(TAK) and STRESS, respectively). mRNA data are normalized by tubulin. Data represent the mean ± SEM (n = 5 to 6 per group). One-way analysis of variance followed by Newman–Keuls *post hoc* test. versus control *P < 0.05 ; ^#^*P* < 0.05 versus stress.

To elucidate the possible role of the TLR-4 pathway in the stress-induced accumulation of inflammatory and oxidative/nitrosative stress mediators, we first determined if there are alterations in the NF-κB pathway in the FC of control and stressed animals injected with vehicle or with TAK-242. After 6 hours of stress, the activity of the pro-inflammatory subunit of the nuclear factor NF-κB p65 did not change compared to control conditions and between both stressed groups of animals (± TAK-242) (Figure 6A). However, IκBα mRNA levels were increased compared to their control (Figure 6B). Finally, pharmacological inhibition of TLR4 in stressed animals was followed by lower levels of IκBα mRNA than stressed animals without TAK-242 (Figure 6B).

**Figure 7:**
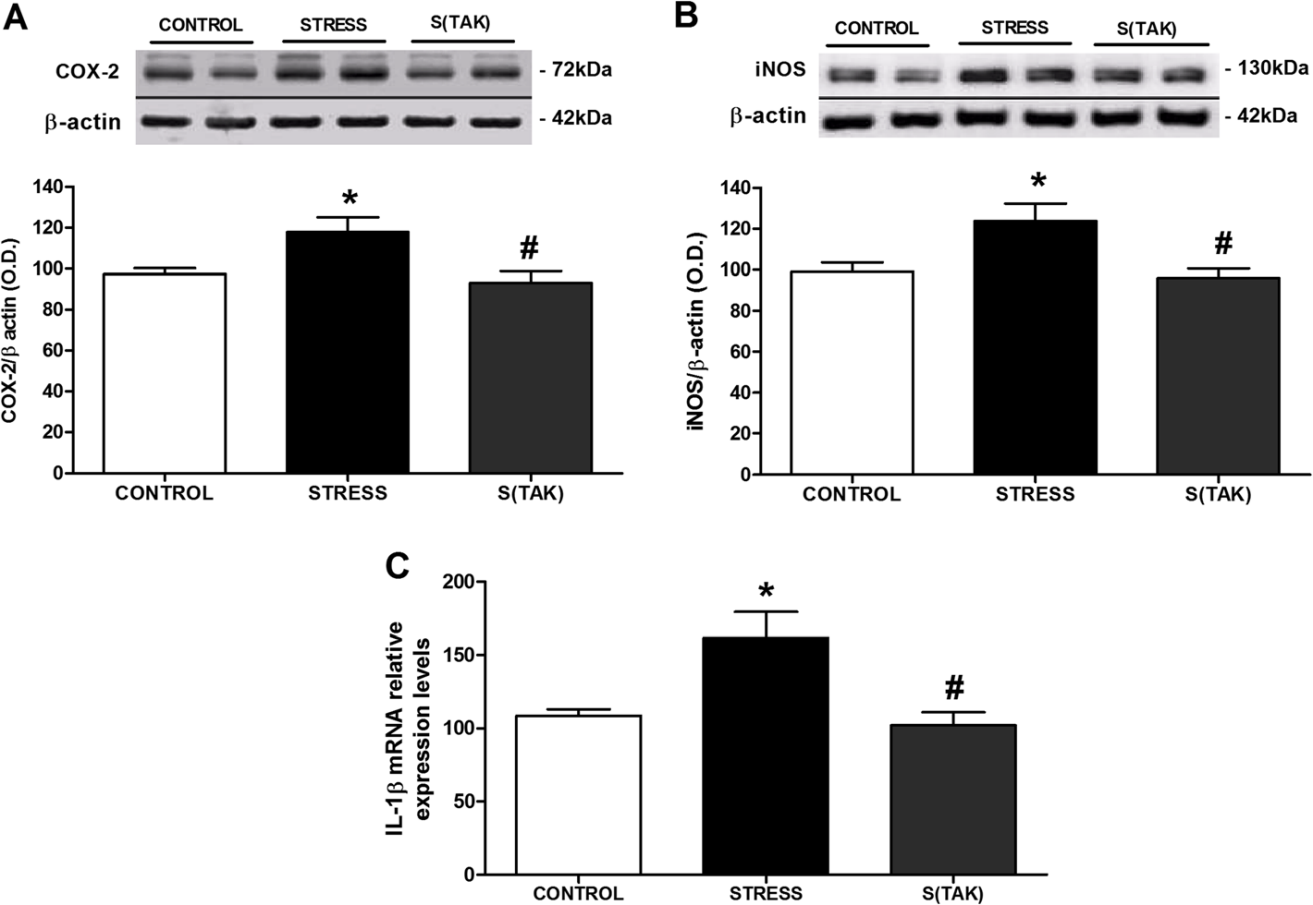
**Anti-inflammatory effects of systemic TAK-242 after stress exposure in rat brain frontal cortex.** Western blot detection of the pro-inflammatory enzymes **(A)** inducible cyclooxygenase (COX-2) and **(B)** inducible nitric oxide synthase (iNOS), and densitometric analysis of the respective bands of interest in brain cortex samples from control (CONTROL) and stressed rats with TAK-242 (S(TAK)) or without TAK-242 (STRESS). The densitometric data of the respective band of interest are normalized by β-actin (lower band). In **A** and **B**, the data are representative of two experiments (n = 3 to 4 per group in each experiment). One-way analysis of variance followed by Newman–Keuls *post hoc* test. *P < 0.05 versus control; ^#^*P* < 0.05 versus stress. O.D., optical density. **(C)** IL-1β mRNA relative levels in brain frontal cortex samples of control and stressed rats with or without TAK-242 (S(TAK) and STRESS, respectively). mRNA data are normalized by tubulin. Data represent the mean ± SEM (n = 4 to 5 per group). One-way analysis of variance followed by Newman–Keuls *post hoc* test. *P < 0.05 versus control ^#^*P* < 0.05 versus stress.

The increase in IκBα mRNA levels could be an autoregulatory mechanism switched on by NF-κB to block its prolonged stimulation, as is the case after 6 hours of stress exposure. Stress exposure also caused an increase in the protein expression of the NF-κB-dependent proinflammatory enzymes COX-2 and iNOS in brain FC (Figure 7A, B). However, under stress conditions, TAK-242 treated animals did not show an increase in the protein expression of these enzymes when compared to their control (Figure 7A, B). Indeed, in the case of COX-2, pharmacological treatment decreased stress-induced increase in COX-2 protein when compared to stressed animals without TAK-242 (Figure 7A).

**Figure 8:**
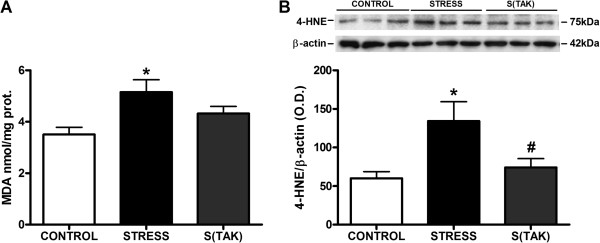
**Antioxidant effects of systemic TAK-242 after stress exposure in rat brain frontal cortex. (A)** MDA and **(B)** 4-hydroxynonenal (4-HNE) protein adduct levels in frontal cortex homogenates of control (CONTROL) and stressed rats with TAK-242 (S(TAK)) or without TAK-242 (STRESS). Data represent the mean ± SEM (n = 5 per group). One-way analysis of variance followed by Newman–Keuls *post hoc* test. *P < 0.05 versus control. ^#^*P* < 0.05 versus stress. O.D., optical density.

In addition, IL-1β mRNA levels in rat brain FC were also determined as a specific pro-inflammatory marker suitable to be affected by the TLR-4 pharmacological modulation. Animals receiving TAK-242 did not show an increase in IL-1β after 6 hours of stress (Figure 7C). As an additional indicator of stress-induced cellular damage that could be affected by TLR-4 selective inhibition, we measured the accumulation of MDA and 4-HNE in the brain FC. Animals treated with vehicle presented MDA over-accumulation after stress exposure that was partly prevented by TAK-242 pre-treatment, although it did not reach statistical significance (Figure 8A). MDA levels of stressed rats without TAK-242 are not different from the control data (*P* > 0.05) (Figure 8A). In the case of 4-HNE, stress produced an over-accumulation of 4-HNE protein adducts in the brain FC that was fully prevented by the administration of TAK-242 (Figure 8B).

## Discussion

The present work shows that acute restraint stress exposure upregulates TLR-4 mRNA and protein expression in rat brain FC. In addition, the administration of its selective inhibitor TAK-242 prevents the neuroinflammation and the accumulation of potentially deleterious oxidative/nitrosative mediators induced by stress exposure, suggesting a possible regulatory role for TLR-4.

The series of experiments using antibiotic intestinal decontamination also suggest a role for bacterial translocation on TLR-4 signalling pathway activation after stress exposure. This relationship has also been shown in sub-chronic and chronic mild stress conditions, using a similar protocol of antibiotic intestinal decontamination [24,37].

Previous studies suggest that TLR-4 is a sentinel receptor that regulates gut barrier permeability. These studies showed that, in certain experimental pathological settings (stress exposure included), TLR-4-deficient animals presented an aggravated intestinal dysfunction, inflammation and subsequent bacterial translocation [37,38]. Conversely, our results did not show any significant effect of TAK-242 on LBP levels and bacterial translocation after 6 hours of stress exposure. Possible explanations could be a reduced bioavailability of TAK-242 at gut level in stress conditions, or the high degree of bacterial translocation present, making a single dose of TAK-242 insufficient to affect this process. In this vein, the great majority of roles proposed for TLR-4 in different experimental settings have been found using TLR-4 knock-out or TLR-4 functional deficient C3H HeJ mice [39] instead of its pharmacological modulation, probably due to the reduced number of potent and specific inhibitors currently available [40].

Although we have demonstrated that bacterial translocation is responsible, at least in part, for the stress-induced TLR-4 upregulation, several other non-excluding mechanisms should not be ruled out, such as the possible involvement of potential signals conveyed by the vagus nerve or the sympathetic nervous system. More specifically, TLR-4 could be regulated by other mediators also activated by stress, such as heat shock proteins [41] and other DAMPs [42]. These molecules are also formed in stress-related disorders in response to LPS activation of TLR-4 and may re-activate TLR-4, thus closing a vicious cycle whose consequences should be investigated. In addition, the excitatory amino acid glutamate that is rapidly released after stress exposure in the rat brain FC [43] is able to regulate TLR-4 via an *N*-methyl-D-aspartic acid-dependent mechanism after the systemic administration of LPS [44]. At the peripheral level, classical stress mediators such as epinephrine/norepinephrine/β_2_ adrenergic receptor and corticotrophin releasing factor also regulate TLR-4 expression [45,46].

The number of studies regarding a direct effect of stress exposure protocols on brain TLR-4 protein expression is reduced because the most commonly used immune/inflammatory stimulus is systemic LPS and its reported effects are at the mRNA level, often being contradictory [47,48]. There are only a few studies checking the effects of restraint stress on TLR-4, all of them showing increased levels of TLR-4 mRNA in the brain FC, spleen and myocardium of chronically stressed rats [24,49,50]. In addition, other authors have previously shown that TLR4 is upregulated in neurons in response to a severe inflammatory challenge, such as ischaemic stress [51] and that TLR-4 signalling influences stress-sensitive behaviours in mice, such as spatial reference memory, fear learning and memory [52].

Our results agree with previous studies also showing TLR-4 expression in astrocytes [53] and microglia [54] under inflammatory conditions. However, further quantitative immunohistochemical studies are needed to identify the nature of the cellular types implicated in the upregulation of TLR-4 elicited by stress exposure and the potential function of TLR-4 present in the FC regulating the hypothalamo-pituitary-adrenal axis response to LPS or other immune/inflammatory challenges.

In addition, the lack of effect of MD-2 and MyD88 under our stress protocol could be indicating that the time course of activation of both proteins is delayed. This possibility should be checked in long lasting stress conditions. In fact, MD-2 is activated after sub-chronic (2 hours/day during 4 consecutive days) and chronic mild stress protocols [24,37].

Regarding MyD88, it is possible that other TLR-4 MyD88-independent signal transduction pathways such as the TRIF-dependent pathway could also be activated by stress. Finally, a plausible interpretation is that TLR4 pathways may have been primed for activation, but are not actually active under these conditions.

To our knowledge, our results showing the effects of TAK-242 preventing the stress-induced accumulation of potentially deleterious pro-inflammatory and oxidative/nitrosative mediators are original. Some authors have reported a similar anti-inflammatory/pro-survival profile but at a peripheral level in *in vivo* endotoxic shock models [55,56] and in *in vitro* macrophage cultures [25].

Based on all these findings, a potential role as adjunctive therapy in severe sepsis and septic shock has been suggested for TAK-242 and other drugs targeting the TLR-4 pathway [57]. However, the result of a clinical trial evaluating the possible use of TAK-242 for the treatment of severe sepsis failed, in terms of reducing the mortality rates in patients [58]. Another clinical trial checking the efficacy and safety of TAK-242 in patients with sepsis-induced cardiovascular and respiratory failure (NCT00633477) is ongoing.

TLRs have also been related to the pathophysiology of diverse neurological diseases (multiple sclerosis, Alzheimer’s disease or stroke [7]), but to our knowledge the great majority of these studies used genetic approaches and there are no data assessing the use of TAK-242 for the pharmacological inhibition of TLR-4. Clearly, further investigation is warranted to determine the utility of TAK-242 to regulate pathological settings with a more moderated innate immune response than the one present in sepsis/endotoxic shock.

The results presented here suggest that TLR-4 represents an important regulatory factor in the physiological response to stress and also support the possibility for pharmacological manipulations of this pathway in order to minimize brain oxidative and inflammatory damage after stress exposure and in stress-related psycho- and neuropathologies. However, the above comments on the negative results of the completed clinical trial perfectly illustrate the necessity for further investigations about the biology of TLRs to address whether the loss or inhibition of TLR-4 is beneficial or predominantly harmful in pathological scenarios of a different nature. It must be considered that TLRs constitute a family of receptors highly conserved between vertebrates with multiple physiological functions, such as the restoration of CNS homeostasis after injury [59], the proper resolution of the inflammatory process [60] or the trigger of fever and other acute phase responses in response to external noxia [61].

Indeed, another limitation of our study is that it is not possible to know if the most relevant role of TLR is at the CNS level, the periphery, or both. Further studies with specific TLR-4 knock-out mice for each compartment/cellular type expressing this receptor are needed to resolve this issue. In addition, complementary studies regarding TAK-242 ability to cross the brain–blood barrier are needed to determine the proper dose and route of administration to completely block the central TLR-4 signalling pathway and to optimize the duration of its effect. In this sense, a recent study has shown that *in vivo* TLR4 inhibition, employing the same dose of TAK-242 (0.5 mg/kg) that we have used here, attenuated the tumour necrosis factor-α, IL-1β and iNOS expression on microglia post-hypoxia [29].

The elucidation of the mechanisms through which peripheral PAMP or DAMP can activate central TLR-4 deserves further investigation. One or more of the following unexplored processes may be taking place in our stress model: 1) circulating leukocytes expressing TLR-4 release inflammatory molecules capable of activating specific brain areas; 2) direct activation of the TLR-4 present in the brain circumventricular organs and other leaky structures, such as the choroid plexus and leptomeninges; 3) direct activation of TLR-4 expressed by endothelial and perivascular cells forming the blood-brain barrier; and 4) activation of the TLR-4 expressed by microglia, astroglia or neurons surrounding brain microvasculature.

## Conclusions

In conclusion, all the data presented here suggest a functional role for TLR-4 in the activation of the immune innate response elicited by acute restraint stress in rats. In addition, our results suggest that the increased bacterial translocation produced by stress exposure could be a reasonable mechanism capable of activating TLR-4.

Finally, the use of TAK-242 and other compounds interfering with the TLR-4 signalling pathway emerge as a potential therapeutic adjuvant strategy for the treatment of some neuropsychiatric diseases, such as depression or chronic fatigue syndrome, characterized by mild neuroinflammation and oxidative/nitrosative damage.

## Abbreviations

4-HNE: 4-hydroxynonenal; BSA: bovine serum albumin; CFU: colony-forming units; CNS: central nervous system; COX-2: inducible cyclooxygenase; DAMP: damage-associated molecular pattern; ELISA: enzyme-linked immunosorbent assay; FC: frontal cortex; GFAP: glial fibrillary acidic protein; IL: interleukin; iNOS: inducible nitric oxide synthase; i.p.: intraperitoneally; i.v.: intravenously; LBP: lipopolysaccharide binding protein; LPS: lipopolysaccharide; MD-2: myeloid differentiation protein-2; MLN: mesenteric lymph node; MyD88: myeloid differentiation factor 88; PAMP: pathogen-associated molecular pattern; PBS: phosphate-buffered saline; PCR: polymerase chain reaction; RT-PCR: real time-polymerase chain reaction; TLR: toll-like receptor.

## Competing interests

The authors declare that they have no competing interests.

## Authors’ contributions

IG contributed to acquisition, analysis and interpretation of the data. BGB contributed to acquisition, analysis and interpretation of data, drafting the manuscript and revising it critically. JLMM contributed to analysis and interpretation of data and revising the manuscript critically. LA and MLGL contributed to acquisition, analysis and interpretation of microbiological data. JRC revised the manuscript critically. JCL contributed to conception and design, drafting the manuscript and revising it critically for important intellectual content. All the authors have read and approved the final version of the manuscript.
